# Restless Legs Syndrome in shift workers: A cross sectional study on male assembly workers

**DOI:** 10.1186/1740-3391-7-12

**Published:** 2009-09-14

**Authors:** Akbar Sharifian, Marjan Firoozeh, Gholamreza Pouryaghoub, Mehran Shahryari, Mohsen Rahimi, Mohammad Hesamian, Ali Fardi

**Affiliations:** 1 Department of Occupational Medicine, Tehran University of Medical Sciences, Tehran, Iran; 2 HSE Department, SAIPA automobile manufacturing company, Tehran, Iran

## Abstract

**Background:**

Restless Legs Syndrome (RLS) is a common neurological movement disorder characterized by symptoms that follow a circadian pattern. Night and rotating shift work schedules exert adverse effects on functions of the human body by disturbing circadian rhythms, and they are known to cause sleep disturbances and insomnia. In this paper, we investigate the possible association between shift work and RLS.

**Methods:**

This cross sectional study was conducted in an automobile manufacturing factory in Tehran, Iran. A total of 780 male assembly workers were recruited in three groups, each with 260 workers: workers on a permanent morning shift (A) and two different rotating shift schedules (B and C) with morning, afternoon and night shifts. We used the international RLS study group criteria for diagnosis of RLS, and the severity scale for severity assessment in subjects with RLS. Self administered questionnaires were used to gather information on age, smoking, work history, medical condition, and existence and severity of RLS symptoms.

**Results:**

The prevalence of RLS was significantly higher in rotational shift workers (15%) than workers with permanent morning work schedule (8.5%). In workers suffering from RLS, we found greater mean values of age and work experience, higher percentages of drug consumption, smoking, and co-morbid illnesses compared with subjects who did not have RLS, although these differences were statistically significant only for age, work experience and drug consumption.

**Conclusion:**

Rotational shift work acts as a risk or exacerbating factor for Restless Legs Syndrome.

## Background

Restless Legs Syndrome (RLS) is a relatively common, under-diagnosed and treatable neurological disorder characterized by an irresistible urge to move the limbs to stop uncomfortable sensory symptoms in the extremities [1]. Typical symptoms involve the legs, but the arms can also become involved [2].

Currently, there is no single diagnostic test for RLS. The disorder is diagnosed clinically by evaluating the patient's history and symptoms. The clinical features of RLS were first fully characterized by Ekbom in the 1940s [1,3]. The most recent RLS diagnostic criteria were developed at a workshop held by the National Institutes of Health with the members of the International Restless Legs Syndrome Study Group (IRLSSG) [4]. Four essential criteria for the diagnosis of RLS are: 1) a compelling urge to move the limbs, usually associated with unpleasant sensation in the legs; 2) symptoms worse or exclusively present at rest, such as lying or sitting 3) the urge to move or unpleasant sensations are partially or totally relieved by movement, such as walking, stretching or rubbing the legs; and 4) symptoms are worse later in the day or at night [5].

Supportive clinical features for the diagnosis include a response to dopaminergic agents, a family history of the disorder, and Periodic Limb Movements (PLMs). PLMs can be characterized by periodic episodes of stereotyped limb movements occurring during sleep which are very common in a variety of sleep disorders including RLS. The existence of PLMs is, however, not necessary or sufficient to make the diagnosis of RLS [2].

In epidemiological studies, very different RLS prevalence rates have been reported. Some discrepancies may be due to the different diagnostic criteria and methodological tools applied and the different targeted populations with varying ethnicities assessed in the surveys. [1,6-8].

A significant number of people worldwide are exposed to shift work as a result of social needs and economic factors. The relationship between work schedules and health is complex and is influenced by characteristics of the work schedule itself as well as the characteristics of the job, the worker, and the work environment [9]. Shift work exerts adverse effects by disturbing circadian rhythms, sleep, and personal life, and may even be an oxidative stressor [10,11]. The association between shift work and several health problems, including reductions in the length and quality of sleep, gastrointestinal symptoms, and cardiovascular diseases is reported in many investigative studies [12-14].

Symptoms of RLS show a characteristic circadian pattern, and intensity of subjective complaints of RLS patients may be modulated by changes in biological markers such as melatonin [15]. Hence, the disturbance of circadian rhythms associated with rotating shift work may affect the prevalence and intensity of RLS symptoms. The main object of our study was to investigate the potential association of shift work with Restless Legs Syndrome.

## Methods

### Study Design

This cross sectional study was conducted in an automobile manufacturing factory located in Tehran (the capital city of Iran). Factory workers cover morning (7 am - 3 pm), afternoon (3 pm - 11 pm) and night (11 pm - 7 am) shifts. Employees assigned to schedule A are required to work only morning shifts whereas employees assigned to schedules B and C follow a rotating shift schedule. Specifically, employees assigned to schedule B rotate on a six week schedule, where they alternate between the morning and afternoon shifts for 5 weeks, and they are required to work the night shifts in week 6. Employees assigned to schedule C rotate on a three week schedule, where they work the morning shifts for week one, followed by the afternoon shifts and night shifts on weeks two and three, respectively.

A total of 1700 assembly workers were working in the factory at the time of sampling, and a sample of 780 male workers (260 workers in each work schedule) was selected from them, by using a simple randomization. No specific exclusion criteria were used. Each subject signed an informed consent document after the goals of the study were fully explained.

The study consisted of self administered questionnaires completed by participants to provide information on the following items: (i) age, (ii) smoking habits, (iii) medical history for co-morbid illnesses (anemia, diabetes mellitus, chronic kidney disease and Parkinson's disease), (iv) consumption history of the drugs which are known to aggravate or induce RLS (antidepressants, sedating antihistamines, dopamine receptor antagonists including anti-emetic and antipsychotic medications), (v) working history, including the number of years employed as a worker in this factory and work schedule. To assess the existence of RLS we used the four minimal IRLSSG clinical criteria, provided in the Background section, as a screening tool in this paper.

All the participants who fulfilled the IRLSSG criteria were also asked to fill in a form that included questions for assessing the existence of PLMs, history of RLS in their first degree relatives, sleep disturbance of their bed partners due to RLS and the IRLSSG rating scale [16]. Severity of RLS can be classified as mild (≤10 in IRLSSG rating scale), moderate (11-20), severe (21-30) and very severe (31-40).

### Data analysis

For statistical analysis, we used SPSS 11.5 for Windows. Descriptive statistics were used to characterize the study population. Differences between mean values of continuous variables (age and years of service) were tested using *t *tests, while proportions for dichotomous variables were calculated with chi-square and Fisher exact tests. Association of RLS with working schedule was examined by binary logistic regression. Odds ratios with the corresponding 95% confidence intervals were calculated after adjustment for Age and drug consumption. A P-value of less than 0.05 was considered to be significant.

## Results

Twelve participants did not completely fill in the questionnaire, and their data were not included in the analyses. The analysed dataset included 260 workers assigned to group A, 258 shift workers assigned to group B, and 250 shift workers assigned to group C.

Overall prevalence of RLS among the workers was 12.8% (n = 98). Among workers classified as having RLS, 69 (70.4%) reported having PLMs, and 19 (19.4%) had family history of RLS in their first degree relative. In addition to health problems associated with RLS, existence of RLS also affected the quality of life of the individual's bed partner. In our study 60 out of 98 (61.2%) patients reported sleep disturbance of their bed partners. According to the IRLSSG severity scale, 62 out of 98 (63.3%) subjects indicated a severe or very severe RLS (IRLSSG rating scales greater than or equal to 21).

The percentages of current cigarette smokers, chronic disorders, and drug consumption were higher in RLS patients compared with subjects who did not have RLS, but the differences were not statistically significant for cigarette smoking and chronic disorders. Comparisons of demographic and clinical characteristics of RLS patients and control group (non-RLS workers) are shown in Table 1. Workers in neither group reported having Parkinson's disease.

**Table 1:** Comparison of some variables between Restless Legs Syndrome (RLS) patients and control subjects

**Variable**	**RLS (n = 98)**	**Control (n = 670)**	**P**	**Total (n = 768)**
**Age (year)**				

Mean (SD)	35.2 (5.9)	32.7 (5.8)	<0.001	33 (5.9)

Range	24-50	20-58		20-58

**Work experience (year)**				

Mean (SD)	11.8 (5.3)	8.9 (4.7)	<0.001	9.3 (4.9)

Range	2-25	0.5-27		0.5-27

**Current smokers, n (%)**	19 (19.4)	87 (12.9)	0.115	106 (13.8)

**Chronic disorders, n (%)**				

Anemia	6 (6.1)	24 (3.6)	0.258	30 (3.9)

Diabetes mellitus	4 (4.1)	18 (2.7)	0.51	22 (2.9)

Chronic kidney disease	1 (1)	2 (0.3)	0.336	3 (0.4)

Total	11 (11.2)	44 (6.6)	0.097	55 (7.2)

**Drug consumption, n (%)**				

Antihistamines	16 (16.3)	29 (4.32)	<0.001	45 (5.85)

Antidepressants	6 (6.1)	23 (3.43)	0.248	29 (3.77)

Dopamine antagonist	2 (2)	15 (2.23)	1.0	17 (2.21)

Total	24 (24.4)	67 (10)	<0.001	91 (11.8)

The prevalence of RLS was 8.5% (n = 22 out of 260) in group A, 14.7% (n = 38 out of 258) in group B and 15.2% (n = 38 out of 250) in group C. Comparison of RLS prevalences using chi square test showed significant differences between groups B and A (p = 0.03), groups C and A (p = 0.02), and groups B&C and A (p = 0.01). Thus, the prevalence of RLS was significantly higher in rotational shift workers (groups B and C) than workers with permanent morning work schedule (group A) (see Table 2).

**Table 2:** Relation of shift work patterns with occurrence of Restless Legs Syndrome (RLS)

**Group**	**Suffering from RLS**		**Crude OR [95% CI]**	Adjusted* **OR [95% CI]**
			
	**No**	**Yes**		
**Day workers (Group A), n (%)**	238 (91.5)	22 (8.5)	1.0 Reference	1.0 Reference

**Rotational shift workers, n (%)**				

- Group B	220 (85.3)	38 (14.7)	1.87 [1.07-3.26]^§^	1.82 [1.03-3.21]^§^

- Group C	212 (84.8)	38 (15.2)	1.94 [1.11-3.38]^§^	1.98 [1.12-3.51]^§^

- Groups B and C	432 (85)	76 (15)	1.9 [1.15-3.14]^§^	1.89 [1.13-3.16]^§^

**Notes: **Group B: workers who follow a work schedule which consists of weekly rotation between morning and afternoon shifts for five weeks in a row, followed by one week of night shifts on week six; Group C: workers who follow a work schedule which consists of weekly rotation between morning, afternoon and night shifts; n = number; OR = odds ratio; CI = confidence interval

* This analysis was adjusted by age and drug consumption.

^§ ^P-value < 0.05

Comparisons of RLS severity and weekly occurrence between groups are shown in Table 3. According to a cut-off score of 21 to distinguish subjects with severe and very severe symptoms from moderate and mild ones in severity assessment of RLS, our data reveal that workers categorized as having severe or very severe RLS (severity score ≥ 21) are not more common among rotational shift workers (groups B and C) than among workers with permanent morning work schedule (group A) (chi square test, P = 0.2). Also, the number of rotational shift workers (groups B and C) who suffer from RLS on four or more days a week is not significantly larger than the number of workers with permanent morning work schedule (group A) (P = 0.33).

**Table 3:** Comparison of Restless Legs Syndrome severity and weekly occurrence between groups

**Group**	**Severity Scale**		**Weekly occurrence**	
	
	**< 21**	≥ **21**	**< 4 day**	≥ **4 day**
**Day workers (Group A), n (%)**	11 (50)	11 (50)	13 (59.1)	9 (40.9)

**Rotational shift workers, n (%)**				

- Group B	14 (36.8)	24 (63.2)	16 (42.1)	22 (57.9)

- Group C	11 (28.9)	27 (71.1)	18 (47.4)	20 (52.6)

- Groups B and C	25 (32.9)	51 (67.1)	34 (44.7)	42 (55.3)

**Notes: **Group B: workers who follow a work schedule which consists of weekly rotation between morning and afternoon shifts for five weeks in a row, followed by one week of night shifts on week six; Group C: workers who follow a work schedule which consists of weekly rotation between morning, afternoon and night shifts.

## Discussion

This study is one of very few to investigate the relationship between different shift work patterns and the occurrence of RLS. Among the group of rotational shift workers, we compared two rotational shift patterns having different night shifts. Work during the night shift is much more likely to have adverse effects on health than work during afternoon shifts because it disrupts the circadian organization of the body [17,18]. The main contribution of the current study is the use of the IRLSSG criteria for the diagnosis of RLS and the use of IRLSSG severity scale to assess the severity of RLS for a group of shift workers. Because our subjects had very similar job tasks in the factory, they also had relatively similar socioeconomic status.

Epidemiological surveys have shown that RLS is a common neurological movement disorder. In the reports of previous Asian studies, RLS prevalence rates ranged from 0.1% in a primary healthcare centre population aged 21 years and older in Singapore to 12.1% in a large Korean cohort [1,19,20]. This discrepancy is probably due to the differences in the demographic characteristics and assessment methods used in the different studies. In our study, the overall prevalence of RLS in male assembly workers was 12.8%. Of course, the fact that our study was conducted with a specific population of factory workers, we cannot generalize the prevalence rate to the general population.

Although we found a higher percentage of cigarette smokers among the RLS individuals than in controls, the difference was not statistically significant (P = 0.115). The possible relation between cigarette smoking and increased risk of RLS has also been investigated in previous studies with inconsistent results [21-23]. A reliable explanation for such a discrepancy may be due to the different measurement criteria used to determine smoking habits. We did not separate heavier from lighter smokers; thus, quantitative assessment of cigarette smoking may better clarify the above-mentioned relationship, if it exists.

Similarly, although we found higher percentages of chronic disorders among RLS individuals than among controls the differences were not statistically significant (see Table 1). The process of assessment of co-morbid illnesses, in our study, was performed through participants' self reports and was not verified with laboratory tests. Thus, there is a possibility that the prevalence of co-morbidity is underestimated in our study and the results do not allow us to infer causality.

We found a greater percentage of antihistaminic and antidepressant drug consumption in RLS individuals than in subjects who did not suffer from RLS, as previously noted by others [24]; however, only the difference in antihistamine consumption was statistically significant.

We found a statistically significant association between rotational shift work and RLS, especially in the group that included more night shifts (Group C) (see Table 2). The percentage of workers suffering from RLS was about twice as high in rotational shift workers (groups B and C) than in day workers (group A). In an epidemiological survey in central Greece, the percentage of shift workers was higher in RLS individuals than in individuals who did not suffer from RLS, although the difference was not statistically significant [23]. In another study that investigated the association between shift work and sleep disorders among police officers from Buffalo, New York (that did not use IRLSSG criteria), the prevalence of RLS was 134 percent higher among women night shift workers than among women workers on other shifts, although the difference was not statistically significant after adjustment for other covariates, and among men there was not any association between night shift work and RLS [25]. In an epidemiological study conducted in five European countries with the aim of documenting the prevalence of RLS and PLMs in the general population and identifying factors associated with these conditions, authors reported that the prevalence of RLS was 5.5% and the prevalence of PLMs was 3.9%, while 18.5% of RLS subjects also had PLMs. Shift or night work was significantly associated with PLMs (OR = 1.4; 95 percent CI = 1.06 - 1.88; P < 0.05) but not with RLS (OR = 0.72; 95 percent CI = 0.50 - 1.05) [26].

Several lines of evidence implicate a dopaminergic pathology in RLS, the strongest of which comes from the pharmacological response of RLS symptoms to dopaminergic agents and aggravation of its symptoms by dopamine blocking type agents [27-29]. Also, it has been reported that higher levels of dopamine and related compounds are found in cerebro-spinal fluid (CSF) in the day time than at night time, with a peak in dopamine at 10 a.m. and a peak for homovanillic acid (HVA) at 2 p.m. [30]. Such circadian variation is consistent with the circadian pattern of RLS symptoms aggravation. Hence, the association between rotational shift work and RLS may be mediated by disruption of circadian rhythmicity.

The role of oxidative stress in the pathological process underlying Parkinson's disease has been reported in other studies [31,32]. Because of similar features in RLS and Parkinson's disease, such as dopaminergic dysfunction and response to dopaminergic agents, it has been suggested that these two diseases may share a common pathophysiology [33,34]. Since shift work has been shown to induce oxidative stress in the human body [11,35,36], it is possible that oxidative stress mediates the association between rotational shift work and RLS.

Our results indicate that more work experience is a risk factor for RLS, possibly because of aging, which is a known risk factor for RLS [6,20,37]. To the best of our knowledge, the relationship between work conditions and job tasks with RLS incidence had not been investigated in previous studies. More experience in assembly work and monotonous manual tasks may have a role in the positive correlation observed between work experience and RLS prevalence, although our results do not provide sufficient information to evaluate such a hypothesis.

Future studies investigating the association between shift work and RLS will be strengthened by increases in sample sizes, by objective laboratory assessment of possible co-morbidities known as causes of RLS, by assessment of the duration of symptoms, and by the use of a prospective study design.

## Conclusion

It can be concluded that rotational shift work may act as a risk or exacerbating factor for Restless Legs Syndrome, which is known to have adverse effects on patients' work performance and quality of life [38]. Although effective and safe treatments exist for RLS, most cases remain undiagnosed. Therefore, screening of shift workers for RLS in their routine periodic examination may be valuable. Further research is necessary to corroborate the associations observed in this study.

## Abbreviations

RLS: restless legs syndrome; IRLSSG: international restless legs syndrome study group; PLMs: periodic limb movements; OR: odds ratio; CI: confidence interval; CSF: cerebro-spinal fluid; HVA: homovanillic acid.

## Competing interests

The authors declare that they have no competing interests.

## Authors' contributions

ASH helped with the conception and design of the study, helped to collect the data, and helped to draft the manuscript. MF helped with the conception and design of the study, helped to collect the data, participated in the data analysis and interpretation, and helped to draft the manuscript. GHP and MSH helped with the conception and design of the study and helped to draft the manuscript. MR, MH and AF helped with the conception and design of the study and helped in study coordination and the data collection.
